# A Volume to Be Read and Studied

**Published:** 1918-04-20

**Authors:** 


### PUBLIC HEALTH NURSING.*

### A Volume to be Read and Studied.

If the growth of the Public Health Nursing Service has been one of almost mushroom rapidity, that is not because it contains elements of impermanency. It was, in fact, a natural but delayed corollary of the Act of 1870 in England, and throughout the world the concomitant of every establishment of compulsory national education. The old prejudice that the animal spirits of children proved super-abundant health and merely needed repression, carried out in some generations by vigorous starving and beating, died hard. Mrs. Whetham, in "The Upbringing of Daughters," has lately retold the story of an energetic board of managers who, some twenty-five years ago, engaged a nurse on their own account to attend to a percentage of really delicate children, and were rapped over the knuckles for their pains as wasters of time given for instruction, by their superior authority. That authority was in a few years to urge upon the attention of all managers the evident fact that instruction is wasted if given to a child not in a condition to receive it. If the tired waves broke vainly for some time without gaining more than inches, however, the main tide of changed opinion has effectively come in, and the need is rather now for a wise understanding of situation and conditions on the part not only of administrators but of that general public from whom can be drawn the volunteer workers, who are not less essential than the professional and expert to the execution of great schemes. Hitherto a comprehensive survey could only be gained by a good deal of promiscuous study, some of the best available information being buried between the covers of soon-forgotten quarterlies. Few within the nursing profession, and not many outside it, have time for such study and research, and the time was more than ripe for such a book as is now offered by Miss M. S. Gardner, President 1913-1916 of the National Organisation for Public Health Nursing in America.

It would be difficult to overpraise this excellent volume, and we can only hope that its title will not cause it to be supposed a manual for students or a dictionary for experts, or that the idea that it hails from America will lessen its success in England. As regards this last point, the fact that we have just welcomed American medical units on their arrival to help in the field should

* *Public Health Nursing.* By Mary Sewall Gardner. (New York: The Macmillan Company. 1917. Price 5s.)

**PUBLIC HEALTH NURSING—(*continued*).**

lend value to information and opinions from the same quarter. Criticism may suggest emendation in a future edition of some trivial matters, such as incorrect French names, and French titles given to Italian institutions, or the mention of the *London Gazette* as a newspaper. Nothing but praise can be given to the presentation of the whole subject, from the sketches of the rise and progress of the various kinds of public Nursing Service—school, domiciliary, industrial, mental, tuberculosis—to the exposition of present conditions and the outlook in each department. Miss Gardner writes not only with ample knowledge, but with an insight that goes to the roots of each question touched, and the wide sympathy that is born of understanding. If she does not suffer fools gladly she realises that the wise must often have dealings with them, and have need to see to it that such dealings are wise. She also sees that the wise are sometimes too ready to rank among the fools those who are merely uninstructed and may be won to the side of wisdom. It is, indeed, the sound, practical good sense as much as the high aims set forth by this book that constitutes its value. In the nurse herself Miss Gardner values wisdom in its combination of tact, sympathy, self-restraint, and thoughtful organisation above even encyclopædic knowledge. Her standard is naturally a high one. When describing the work of those not highly trained "cottage" nurses who in this country will undertake the mother's usual duties during her disablement, she expresses the view that homes that are isolated and poor require the services of a specially competent nurse in time of need. That, however, may be due to a wide American development of the plan favoured here by the Queen's Jubilee Institute, by which a nurse working alone in a rural district undertakes everything—maternity, infant welfare, domiciliary, school clinics, tuberculosis, and chronic-case work. A chapter devoted to the nurse working alone is extremely valuable, and should be read by all committee managers, insisting as it does on the value of voluntary help to relieve the nurse, who, for instance, may be tempted to give her night as well as her day to some pressing case. A nurse is to realise that she has no business to let herself break down.

The chapters on the Nursing Association and Board of Managers are also very good reading. The chairman who has "nothing to report" should be brought to book. Managers are people of very varied kinds, but all have their distinct usefulness, and if Miss Gardner's counsels prevail all will become properly fitting cogs in the great machine of communal welfare. Similarly, the right adjustment must be made in the nurse's relation to the doctor with a certain reciprocity that will develop her initiative, since the work is not precisely like that of an institution or a private house. It must also enter into her dealings with patients' families, especially those of the tuberculous, the mentally afflicted, and the school child. More stress might perhaps be laid on the advantage of the practice, increasingly adopted in this country, of requiring the presence of a responsible relation at the school health examination. The natural indignation of the mother whose child tells her "the doctor says his eyes are to be taken out and scraped" would have less occasion. The problems set forth in the chapter on Industrial Nursing seem to show little difference between those of America and our own, and we are glad to notice that for the public health nurse Miss Gardner advocates a post-graduate course, even if only of a few months, in social service. The book closes with some sound advice, which would have delighted Miss Nightingale, on records and statistics. Finally, we are not sure whether her statement that this branch of the nursing profession is the least well paid is, or will continue to be, generally true. But regarding the value of the work done, full as it is of varied interest, no praise can be too high.

